# Age affects the dynamic interaction between kinematics and gait stability

**DOI:** 10.3389/fbioe.2024.1370645

**Published:** 2024-07-30

**Authors:** Shengyun Liang

**Affiliations:** ^1^ College of Software Engineering, Shenzhen Institute of Information Technology, Shenzhen, China; ^2^ CAS Key Laboratory of Human-Machine Intelligence-Synergy Systems, Research Center for Neural Engineering, Shenzhen Institutes of Advanced Technology, Chinese Academy of Sciences, Shenzhen, China

**Keywords:** Chinese older adults, gait, center of mass, base of support, region of stability

## Abstract

**Introduction:** It is crucial to comprehend the interplay between the center of mass (CoM) and base of support (BoS) in elderly individuals’ body movements, as it could have implications for fall prevention.

**Methods:** The purpose of this study is to characterize age-related differences using the instantaneous location of the CoM and CoM velocity vector in relation to the dynamically changing BoS during walking. Thirty subjects participated in the experiments. Derivation formulas of feasible stability region and age-related statistical analyses were proposed.

**Results:** The stability margin and distance to centroid for elderly group were found to be significantly different from the young group (*p* < 0.05). At heel strike, while the CoMv distance was similar for age-based groups (*p* > 0.05), older individuals demonstrated a greater CoMv distance to the border than the younger at right limb, which suggesting age-related differences in momentum control. In addition, Bland-Altman analysis indicated that the validity was substantial, making it feasible to capture stride-to-stride variability.

**Discussion:** The CoM trajectories and feasible stability region could provide a better understanding of human momentum control, underlying mechanisms of body instability and gait imbalance.

## 1 Introduction

Gait stability is a fundamental concern in the control of human movements. Given this recognition, two primary questions arise: what specific conditions must be met in order to maintain balance, and to what extent is balance achieved in a given situation? Traditional static stability control theory holds that walking instability occurs when the body’s center of mass (CoM) exceeds the base of support (BoS) (Hof et al., 2005). BoS is the area accessible by the plantar center of pressure (CoP) and is considered the stability limit. When external interference occurs, the restoring stability strategy moves the CoM inside the BoS through the fine-tuning effect of the CoP (Dario et al., 2017). Recently, some researchers have proposed and developed the dynamic stability control theory (Lugade et al., 2011). The CoM velocity could be an index to evaluate human stability rather than considering the CoM position and BoS interaction during a quiet stance.

The achievement of stable gait is directly related to the position and velocity of CoM at the instant of foot placement (Yang et al., 2009). The CoM position and velocity in relation to the base of support could assess the risk of falls (Pai et al., 2006). The extrapolated center of mass (XCoM) concept was developed, and the shortest distance from the center of gravity to the support polygon was defined (Hof, 2008). The XCoM and stability margin were used as a measure of balance. Fujimoto and Chou (2016) introduced a feasible region of stability (RoS), which is determined by the permissible ranges of the CoM position and velocity relative to the BoS, to assess the likelihood of a fall occurrence. A similar theory was used to analyze the RoS and characterize age-related differences (Liang et al., 2021). While previous studies have examined the relationship between position and velocity of CoM in relation to the CoP or BoS during movement, no research has yet explored this relationship on an individualized basis, considering the dynamically changing BoS (Lugade et al., 2011). The instantaneous position and velocity of the CoM vector in relation to the dynamically changing BoS could offer insights into how dynamic balance is maintained during gait.

The main contributions of this work are threefold: 1) Methodological innovation: Unlike previous studies, which have typically derived the CoM position using a labeled full-body model in Nexus software (Opti track, 2017), we have instead calculated the CoM based on the Chinese inertial parameters of the adult human body (GB/T17245, 2004). This approach allows for a more accurate representation of the CoM position for Chinese people. 2) Examination of age-related differences: We have examined the trajectory of the CoM in relation to the dynamically changing BoS in healthy young and older adults. Furthermore, we have clarified associations and agreements of motion analysis and characterized age-related differences using a time-independent RoS derived from CoM velocity during gait. 3) Application potential: The time-independent RoS has been bench-marked for potential future fall prevention applications. Additionally, we have collected synchronized motion capture camera images and a single-camera video dataset of movement sequences for both older and younger individuals, providing a valuable dataset for further analysis.

In summary, the objective of this study is to investigate age-related differences in CoM and BoS interactions and to establish a time-independent RoS based on CoM velocity during gait. The underlying hypothesis is that stable human gait is achieved when the direction of motion aligns with stability.

## 2 Materials and methods

### 2.1 Experimental setting

**FIGURE 1:**
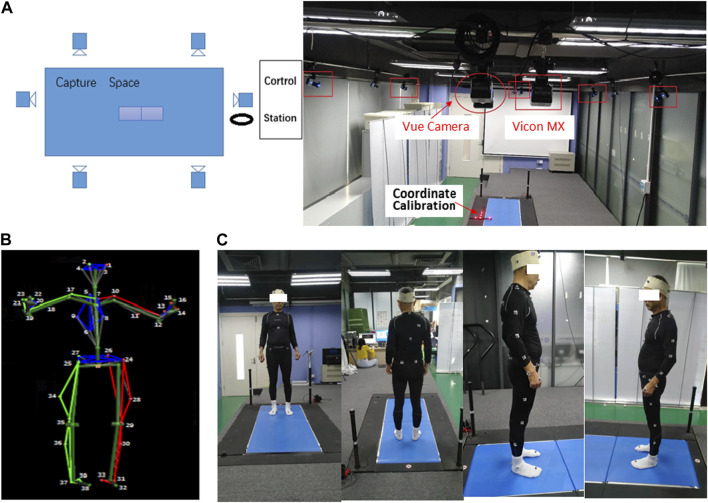
**(A)** Overview of the experiment environment and setup, **(B)** Body39 joints based on the plug-in Gait full-body model, and **(C)** sample image from four viewing angles.

Our experimental setup, depicted in Figure 1A, provided us with the capability to capture data from seven sensors: six Vicon MX motion capture cameras and one Vue video camera. The designated laboratory space was approximately 5 m × 8 m × 3 m, ensuring that the participants were fully visible to all cameras. The motion capture cameras were mounted on wall shelves, with four cameras positioned on each side of the laboratory and two roughly midway along the horizontal edges. These cameras captured three-dimensional marker trajectories at a frequency of 60 Hz. The video camera was also mounted on a wall shelf, recording images of the walking sequences at 60 Hz. The video camera captured RGB files with a resolution of 1920 × 1,080 pixels. We used hardware synchronization techniques to ensure synchronization between the multiple infrared cameras and the digital video camera. This ensured that each point in the motion capture data corresponded to a specific time point in the video frames.

The motion capture data were captured by tracking 39 markers on the human body using a plug-in gait full-body model in Nexus software. Figure 1B shows the Body39 joints labeled by this model. The 39 keypoints are 1: LFHD, 2: RFHD, 3: LBHD, 4: RBHD, 5: C7, 6: T10, 7: CLAV, 8: STRN, 9: RBAK, 10: LSHO, 17: RSHO, 11: LUPA, 18: RUPA, 12: LELB, 19: RELB, 13: LFRA, 20: RFRA, 14: LWRA, 21: RWRA, 15: LWRB, 22: RWRB, 16: LFIN, 23: RFIN, 24: LASI, 25: RASI, 26: LPSI, 27: RPSI, 28: LKNE, 34: RKNE, 29: LTHI, 35: RTHI, 30: LTIB, 36: RTIB, 31: LANK, 37: RANK, 32: LTOE, 38: RTOE, 33: LHEE, and 39: RHEE (Vicon^®^, 2002).

The data for this study were collected through experiments conducted on 15 healthy older adults [Age mean (SD): 56.6 (2.53); BMI mean (SD): 24.168 (2.81)] and 15 healthy younger adults [Age mean (SD): 26.857 (4.63); BMI mean (SD): 21.6 (2.12)]. None of the participants had a history of neurological disease, musculoskeletal issues, traumatic brain injury, visual impairment, or experience of accidental falls. Before the experiment began, all participants were provided with written and oral instructions on the experimental process. Prior to the test, written consent was obtained from each subject to ensure they understood that they had the unconditional right to stop the experimental process at any time during the actual data collection process. This was done to ensure the ethical treatment of the participants and to comply with research ethics guidelines. The participants wore minimal and close-fitting clothes during the experiments. Figure 1C displays sample images captured from four different viewing angles. With their consent and after instrumentation, the participants undertook two 5-s static calibration trials. They stood up straight, with their feet shoulder-width apart, their heads facing forward, and their arms abducted. They then went on to complete five trials of the walking task.

### 2.2 Data processing

#### 2.2.1 Center of mass, CoM

The center of mass was calculated based on the inertial parameters of the adult human body according to the Chinese national standards (GB/T17245, 2004), which are standard for human body measurement. The CoM can be used to analyze and predict body dynamics, defined as follows:

$$CoM=\sum p_{s}*\left[\left(1-l_{s}\right)*a_{u}+l_{s}*a_{l}\right]/\varepsilon ,$$ (1)

where $p_{s}$ is calculated as segment weight divided by the total body weight, $l_{s}$ represents the proportion of the segment’s length above the centroid, and $a_{u}$ and $a_{l}$ are the three-dimensional coordinates of markers attached to specific anatomical landmarks on the body. ε denotes a correction factor ( ε = 0.999 for males and ε = 1.0001 for females). Table 1 provides a list of the values for the parameters used in the CoM analysis.

**TABLE 1:** Anthropometric data.

Segment	Marker- $a_{u}$	Marker- $a_{l}$	Gender	$P_{s}$	$l_{s}$
Head and neck	1,2,3,4	5,7	Male	0.0862	0.469
			Female	0.082	0.473
Upper trunk	5,7	6,8,9	Male	0.1682	0.536
			Female	0.1635	0.493
Lower trunk	6,8,9	24,25,26,27	Male	0.2723	0.403
			Female	0.2748	0.446
Thigh	24,26	28,29 (Left)	Male	0.1419	0.453
	25,27	34,35 (Right)	Female	0.141	0.442
Shark	29	30,31 (Left)	Male	0.0367	0.393
	35	36,37 (Right)	Female	0.0443	0.425
Foot	31	32,33 (Left)	Male	0.0148	0.486
	37	38,39 (Right)	Female	0.0124	0.451
Upper arm	10	11,12 (Left)	Male	0.0243	0.478
	17	18,19 (Right)	Female	0.0266	0.467
Forearm	12	13,14,15 (Left)	Male	0.0125	0.424
	19	20,21,22 (Right)	Female	0.0114	0.453
Foot	14,15	16 (Left)	Male	0.0064	0.366
	21,22	23 (Right)	Female	0.0042	0.349

#### 2.2.2 Base of support, BoS

**FIGURE 2:**
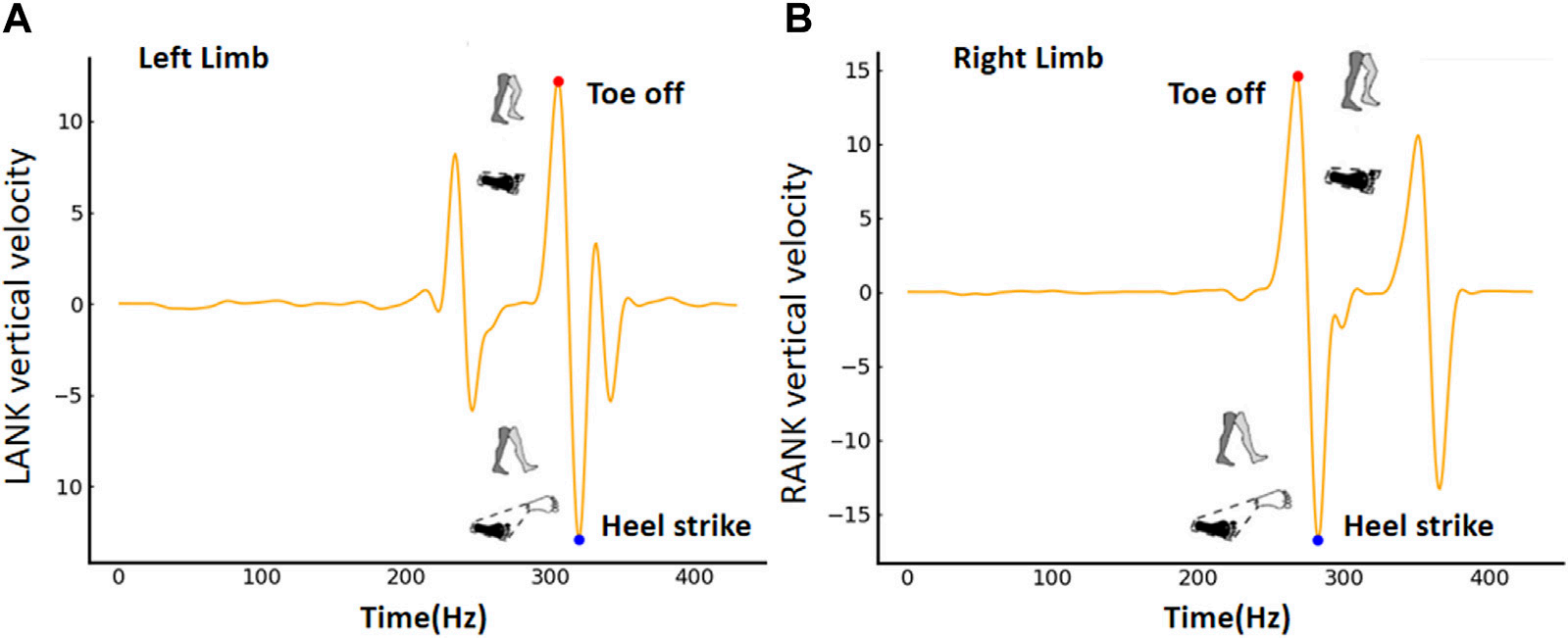
The base of support is determined based on toe off and heel strike for the **(A)** right limb and **(B)** left limb.

The base of the support area was defined based on the configurations of both feet at different stages of the gait cycle, such as heel strike (when the heel first contacts the ground), foot flat (when the foot is fully on the ground), heel off (when the heel lifts off the ground), and toe off (when the toes lift off the ground). During single-limb support, the boundaries of the BoS were determined by the position and orientation of the supporting limb, particularly the foot on the ground. The three-dimensional coordinates of LANK, LTOE, LHEE, RANK, RTOE, and RHEE markers constructed the boundary. During double-limb support, the BoS was defined similarly to single-limb support, encompassing the portions of each foot in contact with the ground, as well as the area between the feet. Figure 2 shows that the BoS area was calculated throughout the gait cycle. Toe off and heel strike were detected based on the vertical velocity of LANK or RANK, respectively. The shaded regions of the foot and the dashed lines, respectively, symbolized the contact area of the foot with the ground and the boundary of the BoS.

At heel strike (CoM inside BoS), the BoS was a triangle. Given any three segments $\vec{U}$ , $\vec{T}$ , and $\vec{S}$ , the test to determine whether they form a triangle is as follows:

$$\left|\vec{U}\right|+\left|\vec{T}\right|>\left|\vec{S}\right|.$$ (2)

The segments were constructed by the three-dimensional coordinates of LANK, LTOE, and LHEE or RANK, RTOE, and RHEE. For example, the three links are represented as $\vec{U}=\text{LANK}-\text{LTOE }\left(\text{or }\vec{U}=R\text{ANK}-R\text{TOE}\right)$ , $\vec{T}=\text{LTOE}-\text{LHEE }\left(\text{or }\vec{T}=R\text{TOE}-R\text{HEE}\right)$ , and $\vec{S}=\text{LHEE}-\text{LANK }\left(\text{or }\vec{S}=R\text{HEE}-R\text{ANK}\right)$ .

At toe off (CoM outside BoS), the BoS was a polygon. Given any four segments $\vec{U}$ < $\vec{T}$ < $\vec{S}$ < $\vec{K}$ , the test to determine whether they form a polygon is as follows:

$$\left|\vec{U}\right|+\left|\vec{T}\right|+\left|\vec{S}\right|>\left|\vec{K}\right|.$$ (3)

The segments were constructed by the three-dimensional coordinates of RANK, LANK, LTOE, and LHEE or LANK, RANK, RTOE, and RHEE. For example, the four links are represented as $\vec{U}=\text{LANK}-\text{LTOE }\left(\text{or }\vec{U}=R\text{ANK}-R\text{TOE}\right)$ , $\vec{T}=\text{LTOE}-\text{LHEE }\left(\text{or }\vec{T}=R\text{TOE}-R\text{HEE}\right)$ , $\vec{S}=\text{LHEE}-R\text{ANK }\left(\text{or }\vec{S}=R\text{HEE}-L\text{ANK}\right)$ , and $\vec{K}=\text{RANK}-\text{LANK }\left(\text{or }\vec{K}=\text{LANK}-\text{RANK}\right)$ .

#### 2.2.3 Region of stability, RoS

The stability measures utilized in the present study were primarily based on the position of the CoM or XCoM relative to the BoS during a specific phase of the gait cycle (e.g., heel strike or toe off). The XCoM considered the position and velocity of the CoM and served as a basis for determining the gait stability requirements. To calculate the XCoM, the formula provided by Hof (2008) was employed:

$$XCoM=CoM+\frac{{CoM}_{v}}{\sqrt{g/l}}.$$ (4)

As Table 2 shows, the CoM represents position, and the CoMv represents velocity. The acceleration of gravity $g=9.81m/s^{2}$ , and l is maximum height of the CoM. Because BoS was confined based on the configurations of both feet, we assume that $X_{h}\leq CoM+{CoM}_{v}/\omega_{0}\leq X_{t}$ . The following formula can be obtained:

$$-\tilde{X}\leq \tilde{{CoM}_{v}}\leq 1-\tilde{X,}$$ (5)

where $\tilde{X}$ and $\tilde{{\text{CoM}}_{v}}$ are the normalized CoM position and velocity, respectively. $\tilde{X}=\left(CoM-X_{h}\right)/L_{f}$ , $\tilde{{CoM}_{v}}={CoM}_{v}/\left(\omega_{0}L_{f}\right)$ , and ${L_{f}=X}_{t}-X_{h}$ $\omega_{0}=\sqrt{g/l}$ . When performing walking actions, subjects may exhibit a swaying motion, and $X_{t}$ and $X_{h}$ indicate the heel and toe position, respectively. Table 2 shows the CoM and BoS interaction variables.

**TABLE 2:** CoM and BoS interaction variables.

Abbreviation	Description
T_con	Time to contact
CoM	CoM position
CoMv	CoM velocity
BoSarea	Area of the BoS
DCoMc	Distance from the CoM to the centroid of the BoS
DCoMs	Shortest distance from the CoM to the boundary of the BoS
DCoMv	Distance from the CoM to the BoS along the direction of the CoMv

### 2.3 Statistical analysis

To assess the CoM-related parameters, the root-mean-squared (RMS) error was calculated. Python (version 3.7) was utilized to investigate the disparities in estimation errors among the parameters. When the variable satisfied both normal distribution and homogeneity of variance, the independent-sample t-test was utilized. However, if the variable failed to meet these criteria, the Mann–Whitney nonparametric test was employed for the comparison analysis. The *p*-value was corrected using Bonferroni multiple comparison. A *p*-value less than 0.05 was considered statistically significant.

To delve deeper into the accuracy and precision between automatic and device-detected variables, the Bland–Altman limits of agreement (LoA) method was employed. This analysis assessed the concordance between the model and the gold standard reference by gauging the accuracy and precision of the tested method. Accuracy was determined by calculating the mean difference (or bias) between the two sets of values (estimated and reference), while precision was determined by calculating the LoA, which represents 95% of the differences, and tracking the spread of the measurement points with respect to those limits. If the following three conditions were met simultaneously (data behavior is good), the difference evaluation of the Bland–Altman method was used to evaluate consistency; otherwise, the ratio evaluation of the Bland–Altman method was used to evaluate consistency. It could indicate that the difference has no proportional bias, that is, the univariate linear regression *p* > 0.05; the variance of the difference is homogeneous, that is, the one-way ANOVA *p* > 0.05; or the difference is normally distributed, that is, the normality test *p* > 0.05.

We could also calculate the mean difference (¯d) or mean ratio (¯r) between automatic and device-detected variables, as well as the 95% LoA (¯d ± 1.96 SD, or ¯r ± 1.96 SD) and the 95% confidence interval (CI) of the 95% LoA. Both the 95% LoA and its 95% CI, located inside the acceptable range of LoA, indicated that the data detected by the two methods were consistent.

## 3 Result

### 3.1 Limb-related differences using CoM and BoS interaction

Mean and standard deviations for selected features for the left limb and right limb are shown in Table 3. A larger BoS area was evident during heel strike, and interestingly, the younger group exhibited significantly higher values than the older group. The stability margin and distance to the centroid were similar between the left limb and the right limb. Nevertheless, notable disparities emerged as the corresponding metrics in the younger group were significantly larger than those of the older group. In addition, there was a significant difference in pairwise comparisons of DCoMv at heel strike. It has been observed that the right limb is primarily utilized for propulsion, whereas the primary function of the left limb is to maintain the stability of the body and contribute slightly to propulsion. In experiment, all participants were right-footed (Wikipedia, 2001), i.e., they all preferred to use right leg when playing football.

**TABLE 3:** Age group averages (SD) for the CoM and BoS interaction at heel strike and toe off.

Variable	Older		Younger	
	Left limb	Right limb	Left limb	Right limb
T_con	14.200 (1.014)	14.400 (1.183)	14.000 (1.198)	14.461 (1.000)
At heel strike (CoM inside BoS)				
BoSarea	160.232 (23.107)	165.682 (22.348)	179.300 (26.828)	181.331 (37.956)
DCoMc	0.835 (0.057)	0.841 (0.057)	0.904 (0.045)	0.905 (0.041)
DCoMs	0.777 (0.059)	0.776 (0.060)	0.834 (0.037)	0.836 (0.036)
DCoMv	1.198 (0.749)	2.074 (1.979)	1.482 (2.096)	1.755 (1.419)
At toe off (CoM outside BoS)				
BoSarea	29.779 (5.983)	30.761 (6.273)	30.412 (4.146)	33.382 (7.193)
DCoMc	0.860 (0.059)	0.859 (0.059)	0.949 (0.069)	0.941 (0.063)
DCoMs	0.836 (0.058)	0.839 (0.062)	0.923 (0.065)	0.918 (0.059)
DCoMv	5.427 (1.548)	5.434 (0.755)	6.714 (1.745)	6.486 (1.525)

The functions of the left and right limbs were distinct, and there was evidence to suggest the necessity of discussing them separately when analyzing the differences between the older and younger groups. Given the asymmetry and specialized roles of each limb in various motor tasks, it was crucial to consider their individual performance in order to gain a comprehensive understanding of the impact of age on lower limb function. By analyzing the data for the left and right limbs separately, we could more accurately assess the differences in stability and centroid distance between the younger and older groups and thereby gain deeper insights into the aging process and its effects on limb function.

### 3.2 Age-related differences using CoM and BoS interactions

**FIGURE 3:**
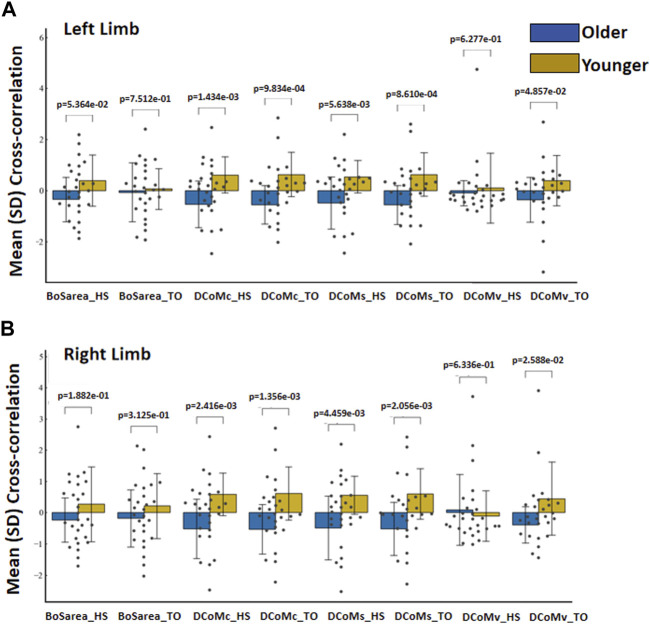
Mean (SD) cross-correlation of CoM and BoS at heel strike and toe off between age groups for the **(A)** left limb and **(B)** right limb.

Statistically significant interactions between the age groups were identified for the area of the BoS (BoSarea), the distance from the CoM to the centroid of the BoS (DCoMc), the shortest distance from the CoM to the boundary of the BoS (DCoMs), and the distance from the CoM to the BoS along the direction of the CoMv (DCoMv) at heel strike and toe off for the left and right limbs (Figure 3). The *x*-axis and *y*-axis represent the variables and values between age groups, respectively. The orange and blue colors represent the younger and older groups, respectively.

The greatest separation between all CoM variables and the BoS was found at the instant of toe off and prior to heel strike. No differences in BoSarea were seen among the older and younger groups at heel strike (*p* = 5.364e−02) or during toe off (*p* = 7.512e−01) at the left limb. The stability margin and distance to the centroid were significantly different between age groups at heel strike (*p* = 1.434e−03; *p* = 5.638e−03) or during toe off (*p* = 9.834e−04; *p* = 8.610e−04) at the left limb. While no differences were seen in the DCoMv during toe off (*p* = 4.857e−02), the younger group demonstrated a greater CoMv distance to the border than the older group at heel strike (*p* = 6.277e−01) at the left limb.

Similarly, no differences in BoSarea were seen among the older and younger groups at heel strike (*p* = 1.882e−01) or during toe off (*p* = 3.125e−01) at the right limb. The stability margin and distance to the centroid at heel strike were significantly different between the age groups (*p* = 2.416e−03; *p* = 4.459e−03) or during toe off (*p* = 1.356e−03; *p* = 2.056e−03) at the right limb. While no differences were seen in the DCoMv during toe off (*p* = 2.588e−02), the younger group demonstrated a smaller CoMv distance to the border at heel strike than the older group (*p* = 6.336e−01) at the right limb.

### 3.3 RoS boundary analysis between age groups

**FIGURE 4:**
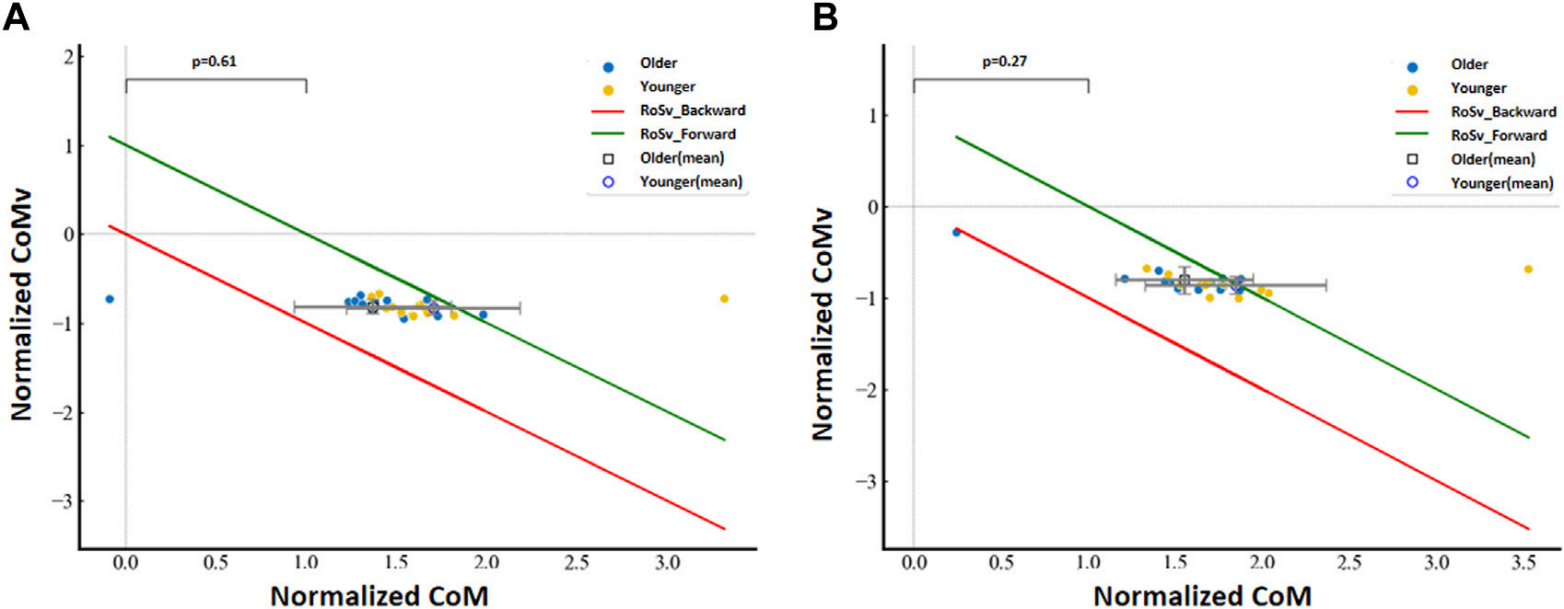
Normalized CoM velocity with respect to the normalized CoM position between age groups for the **(A)** left limb and **(B)** right limb.

Regions of stability defined with CoM and CoMv are constructed in Figure 4. The horizontal axes are the normalized CoM positions, and the vertical axes are the normalized CoM velocities. The RoS boundaries are represented by lines, and the scattered points show the data from each participant, with different shapes denoting the two groups.

No significant group differences were detected for the normalized CoM velocity with respect to the normalized CoM position for the left limb (*p* = 0.27) and the right limb (*p* = 0.61). However, we found that data at the left limb from younger subjects were 20.076% (3 of 13 subjects) located outside the boundary of the RoS, and data from older subjects were 6.667% (1 of 15 subjects) located outside the boundary of the RoS. We also found that data at the right limb from younger subjects were 7.692% (1 of 13 subjects) located outside the boundary of the RoS, and data from older subjects were 13.333% (2 out of 15 subjects) located outside the boundary of the RoS.

### 3.4 Comparison between automatic and device-detected variables

Separately, 112 sets of valid DCoMc, DCoMs, and DCoMv data were obtained. Based on the results of univariate linear regression (*p* > 0.05), one-way ANOVA (*p* > 0.05), and normality testing (*p* < 0.05), those could not meet the data behavior test. Therefore, the ratio evaluation of the Bland–Altman method was used to assess consistency, as shown in Table 4.

**TABLE 4:** Consistency analysis between automatic and device-detected variables.

Variable	Data behavior test (p-value)			Bland–Altman consistency test				
	Linear regression	One-way ANOVA	Normality test	¯r	¯r CI	95% LoA	Lower LoA CI	Upper LoA CI
DCoMc	0.999	0.978	<0.001	0.89	(0.68, 1.10)	(−1.33, 3.11)	(−1.54, −1.11)	(2.90, 3.32)
DCoMs	0.999	0.998	<0.001	0.80	(−0.16, 1.77)	(−9.28, 10.89)	(−10.24, −8.32)	(9.93, 11.85)
DCoMv	0.999	0.051	<0.001	1.41	(0.20, 2.61)	(−11.19, 14.00)	(−12.39, −9.99)	(12.80, 15.21)

**FIGURE 5:**
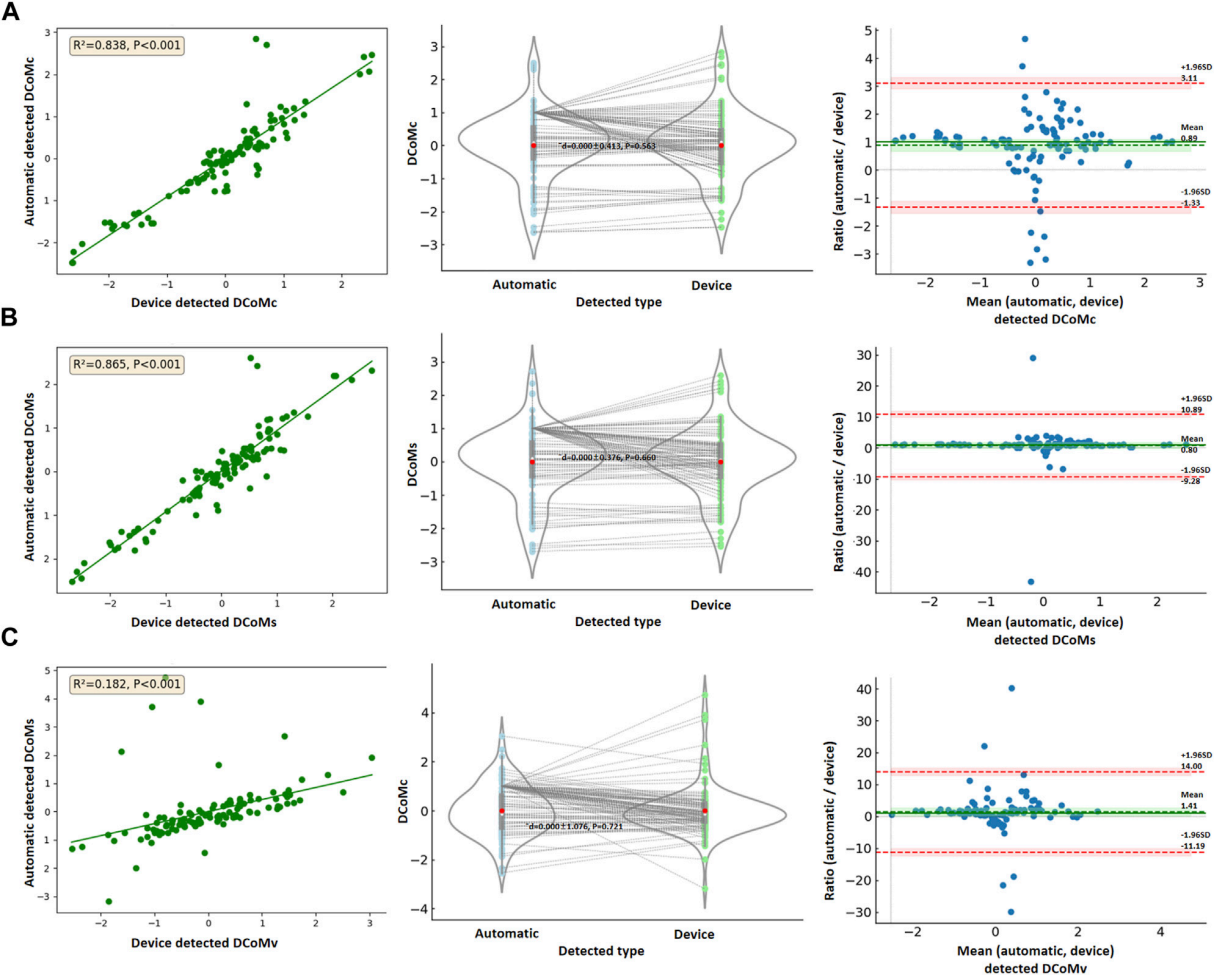
Data comparison between automatic and device-detected **(A)** DCoMc, **(B)** DCoMc, and **(C)** DCoMs.

The automatic and device-detected DCoMc, DCoMc, and DCoMs were significantly correlated (*p* < 0.001). There was no statistically significant difference between the two methods in the DCoMc (¯d = 0.000 ± 0.413, *p* = 0.563), DCoMs (¯d = 0.000 ± 0.376, *p* = 0.660), and DCoMv (¯d = 0.000 ± 1.076, *p* = 0.721), as shown in Figure 5. The mean ratios (¯r) of DCoMc, DCoMs, and DCoMv were 0.89, 0.80, and 1.41, respectively. The 95% LoA and 95% confidence intervals (CI) for each variable are presented in Table 4. Bland–Altman plots were created, with the red dashed lines representing the upper and lower limits of the 95% LoA and the green dashed lines indicating the mean ratio. In the Bland–Altman plots corresponding to each variable, data points almost fall within the orange dashed lines, indicating that the 95% LoA falls within the acceptable range of LoA. This suggested that the data measured by the two methods show good agreement, as shown in Figure 5.

## 4 Discussion

The main findings of this study show that the proposed methods for characterizing age-related differences using CoM and BoS interaction, as well as constructing time-independent RoS using CoM velocity during gait, were robust. Several previous studies have used RoSv boundaries analysis to study the gait stability of walking or other human movements (Fujimoto and Chou, 2016; Liang et al., 2021). Our findings were consistent with these reports in that CoM and BoS interaction provided promising quantitative information about human movement.

Falls were the most common of all accidents, with approximately 50% occurring during walking. Defining BoS during gait can further reveal the application of foot placement strategies that aim to capture dynamically changing CoM to prevent falls (Winter 1995). A quantitative definition of the BoS was previously established (Delisle, 1998); however, only double-limb support of quiet stand was investigated, and dynamic changes to the BoS and its interaction with the CoM were not studied during gait. Utilizing the technique presented, we found that the stability margin and distance to the centroid were significantly different between age groups at heel strike and during toe off. Past work has also shown that the quantified results of CoM and BoS interaction may be a useful measure during dynamic situations (Macie, 2023), with the projection of the CoM to the supporting boundary being used as a measure of stability among walking machines (Dario, 2016; Felix, 2023).

By applying the XCoM concept, the position and velocity of CoM in relation to the BoS could trigger changes in foot placement for the subsequent step (Devetak, 2019). The RoSv boundaries analysis indicated that the older group demonstrated no significant difference from the younger group. These results suggested that the RoSv represented the velocity-related dynamic stable region during walking, reflecting the subjects’ ability to control CoM. If the direction of motion aligned with the stable region, stability was maintained. The sit-to-walk action can serve as a validation of this assertion (Gao, 2019). We also observed that numerous younger participants were situated beyond the RoSv boundary, as depicted in Figure 4. This observation may be attributed to the fact that younger individuals tended to rely on dynamic inertial control for achieving balance, whereas older individuals were more inclined to utilize a relatively static approach for supporting their balance control. This finding aligned with previous research (Carty, 2011). Reflecting on the actual experimental process, we observed that some subjects initially exhibited instability, prompting them to adapt their strategy to maintain balance. These older individuals displayed varying degrees of instability and resorted to placing their feet down to ensure stability, thereby validating the accuracy of the stability region.

In addition, the data comparison analysis results showed that the automatically detected variables had a high correlation with those detected by traditional devices. These have no noticeable fixed bias (mean ratio ≈ 1) and similar LoA ranges (±1.96SD) for DCoMc, DCoMs, and DCoMv, respectively. This was consistent with the results given by Chebel and Tunc (2023). Although there were differences in the measurement results of some indicators, they were within the allowable consistency range. We found that the traditional device has relatively weak computing power for DCoMc, resulting in many comparison data samples of DCoMc falling outside the LoA range. Excluding abnormal points, the data were within the acceptable range. Combining the above analysis results, it could be considered that the proposed method had a good verification result.

With regard to study limitations, we did not pre-calculate the sample size. Nevertheless, the number of subjects exceeded the typically recommended requirements for reliability studies Koo and Li (2016), as 30 individuals were instructed to walk three times in order to gather sufficient data for the analysis. Our approach involves recording subjects’ images from four different viewing angles using video cameras, allowing us to accumulate a database that can be used to refine gait events. In future work, it may be possible to achieve the CoM-related parameters from video image-based human posture recognition models. It could be beneficial to train models that are tailored to specific populations, such as older individuals who experience accidental falls or abnormal gait patterns. The markerless-based analysis described in the current study holds promise for future application (Liang et al., 2022). This method has the potential to classify different gait types and automatically extract quantitative gait information from a single image.

## 5 Conclusion

This study demonstrated a potential use of the combinations of CoM position and velocity to differentiate individuals according to their control abilities during walking. Given economic and time constraint problems, we gained several insights from this exercise: 1) The age-based difference between the quantified results of CoM and BoS interaction supports our initial hypothesis that human gait is stable if the normalized CoM velocity point is contained with the convex hull of the RoS; 2) the Bland–Altman analysis within the two methods was in almost complete agreement. It was indicated that our RoS boundaries analysis could be used as a quantitative stability assessment of gait outside of a clinic. Future work should address predicting instability or fall risk to help older people who have experienced accidental falls.

## Data Availability

The raw data supporting the conclusion of this article will be made available by the authors, without undue reservation.
